# Nanofibrillation is an Effective Method to Produce Chitin Derivatives for Induction of Plant Responses in Soybean

**DOI:** 10.3390/plants9070810

**Published:** 2020-06-28

**Authors:** Hironori Kaminaka, Chihiro Miura, Yukiko Isowa, Takaya Tominaga, Mamu Gonnami, Mayumi Egusa, Shinsuke Ifuku

**Affiliations:** 1 Faculty of Agriculture, Tottori University, Tottori 680-8553, Japan; cmiura@tottori-u.ac.jp (C.M.); ponta.ttr@gmail.com (Y.I.); egusa-m@tottori-u.ac.jp (M.E.); 2 The United Graduate School of Agricultural Science, Tottori University, Tottori 680-8553, Japan; D20A1007C@edu.tottori-u.ac.jp; 3 Department of Agricultural Science, Graduate School of Sustainable Science, Tottori University, Tottori 680-8553, Japan; m.cl8.purple@gmail.com; 4 Graduate School of Engineering, Tottori University, Tottori 680-8552, Japan; sifuku@tottori-u.ac.jp

**Keywords:** chitin, chitin nanofiber, *Glycine max*, growth promotion, nanofibrillation, transcriptome

## Abstract

Chitin, an *N*-acetylglucosamine polymer, is well-known to have unique biological functions, such as growth promotion and disease resistance induction in plants. Chitin has been expectedly used for improving crop yield using its functions; however, chitin derivatives, such as chitin oligosaccharide (CO) and chitosan, are widely used instead since chitin is difficult to handle because of its insolubility. Chitin nanofiber (CNF), produced from chitin through nanofibrillation, retains its polymeric structure and can be dispersed uniformly even in water. Here, the effects of CO and CNF on plant responses were directly compared in soybeans (*Glycine max*) to define the most effective method to produce chitin derivatives for plant response induction. The growth promotion of aerial parts was observed only in CNF-treated plants. The transcriptome analysis showed that the number of differentially expressed genes (DEGs) in CNF-treated soybeans was higher than in CO-treated soybeans. Notably, the expression patterns of DEGs were mostly similar but were strongly induced by CNF treatment as compared with the CO group. These results reveal that CNF can induce stronger plant response to chitin than CO in soybeans, suggesting nanofibrillation, rather than oligomerization, as a more effective method to produce chitin derivatives for plant response induction.

## 1. Introduction

Chitin is a natural polymer with an *N*-acetylglucosamine-repeating structure, which is a highly abundant polysaccharide occurring mainly in the exoskeletons of arthropods, including crustaceans and insects, and in the cell walls of yeast and fungi [1,2]. Chitin and its deacetylated derivative chitosan are well-known to have various kinds of unique functions that can contribute to the crop yield improvement. Chitin derivatives directly induce defense responses and affect plant growth, as well as stimulate beneficial microbe activity in soils, promoting plant growth and disease resistance [1]. These highly sophisticated functions that increase the crop yield have been expectedly used for chitin-derived materials widely as an ecofriendly promising agent instead of chemicals, such as fertilizers and pesticides, in agriculture. Original polymeric chitin generally produced from crab or shrimp shell wastes is difficult to be insoluble in most solvents mainly because of the highly extended hydrogen-bonded semi-crystalline structure [3]. Instead of original chitin, the water- or weak acid-soluble derivatives, chitin oligosaccharides (CO) and chitosan, have been generally used. However, not only these chitin derivatives, but also chitin, per se, are produced using strong acid/alkali, which are problematic wastes [1]. Therefore, the developing of ecofriendly chitin derivatives, which can reduce the problematic chemical wastes, has recently been trialed.

We have previously developed chitin nanofiber (CNF) produced directly from original chitin powder by physical nanofiber isolation (nanofibrillation) from microfibrils that consist of 2–5 nm diameter nanofibers, without hazardous chemicals [2]. CNF retains its polymeric structure but can be equally dispersed not only in weak acid but also in water, unlike original chitin, and used as a solution. These features of CNF resolve the problems on the difficulty of handling chitins and indicate that chitin nanofibrillation may be a strong solution to develop ecofriendly chitin derivatives. Original chitin is impossible to use for uniform treatment because of its insolubility, but using CNF, the function of polymeric chitin can be elucidated, and the effects of high- and low-molecular weight chitins on organisms can be directly compared.

We have tried to figure out the function of polymeric chitin in plants using CNF by considering the aforementioned advantages. Chitin is well-known as a defense response elicitor and referred to as part of the microbe-associated molecular patterns (MAMPs) [4]. CNF induces reactive oxygen species (ROS) production, which is a hallmark of elicitors, in *Arabidopsis*, rice, tomato, and strawberry [5,6]. The foliar application of CNF to *Arabidopsis* leaves increased disease resistance against bacterial pathogen *Pseudomonas syringae* pv. *tomato* DC3000 and fungal pathogen *Alternaria brassicicola* [5]. CNF supplementation to soils systemically induced disease resistance against fungal pathogens, such as *Alternaria brassicicola* in Arabidopsis and cabbage and *Colletotrichum fructicola* in strawberries [6]. Similarly, the symptoms caused by *Fusarium oxysporum* f. sp. *lycopersici*, the causative agent of Fusarium wilt disease to tomatoes, were suppressed by adding CNF and CNF composites prepared from crab shells to soils [7]. However, CNF, per se, was an unusable nitrogen source for growing tomato plants [8]. CNF application at high concentrations strongly induced disease resistance but simultaneously inhibited plant growth, which may be caused by the tradeoff between growth and defense [6,9]. CNF application under nutrient-limiting conditions also showed a growth-promoting action and significantly increased nitrogen content associated with high nitrogen uptake efficiency in hydroponically cultivated tomato [10]. It is worth noting that CNF-induced ROS production level was higher than that induced by COs (the mixture of DP [degree of polymerization] 2–6, and DP6) in *Arabidopsis* and rice, whereas any significant difference in the effects of CO and CNF was not observed on disease resistance in *Arabidopsis* by foliar application and on growth promotion in tomato [5,10]. The mode of action by CO and CNF seems to be common, but it has been unclear yet whether the degree of those effects is the same or not since the specific function of CNF has not been found in plants so far.

In this study, we aimed to define the most effective method to produce chitin derivatives applicable to improving crop yield by comparing effects of oligomerization and nanofibrillation using CO and CNF, respectively. The direct comparison of CO- and CNF-treated plants was performed in soybeans (*Glycine max*) belonging to Fabaceae (also called Leguminosae) family, which is the third-largest angiosperm family that includes the important commercial crops [11], by considering the application of previous findings to crops that are still unexamined. CNF supplementation to soils without fertilizer promoted the growth of aerial parts, whereas CO treatment did not. Transcriptome analysis was conducted by RNA sequencing (RNA-seq) to examine the comprehensive changes in soybean plants following CO and CNF treatments. The number of differentially expressed genes (DEGs) in CNF-treated soybeans was higher than that in CO treatment. It was notably shown that DEGs expression patterns were mostly similar, but CNF treatment strongly induced their expression, as compared with CO. These results reveal that CNF can induce stronger plant response to chitin than CO in soybeans, indicating nanofibrillation, rather than oligomerization, as the effective method to produce chitin derivatives possibly to exert chitin’s function in plants.

## 2. Results

### 2.1. Effects of Chitins on the Plant Growth in Soybeans

**Figure 1:**
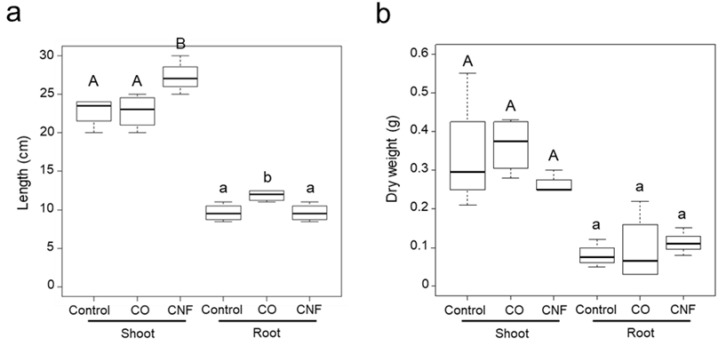
The effects of chitin supplementation to soils on soybean plant growth. The soybean plants were grown on soil mixed with equal volume of 0.01% (w/v) chitin oligosaccharide (CO) solution or chitin nanofiber (CNF) dispersion for four weeks and then harvested for measurement of length (**a**) and dry weight (**b**) of shoots and roots. Data are shown as mean and SE (n = 4). Different letter types (uppercase and lower case) denote significant differences (Tukey’s HSD test, *p* < 0.05). Data are representative of three independent biological experiments, showing similar results.

Soybean plants were grown in soil supplemented with CO and CNF but without any nutrients, because soybeans can be grown only using their own nutrients that are stored in its seeds from germination to the primary growth stage. Using this growth condition, the growth-promoting effects of both CO and CNF associated with nitrogen uptake efficiency, which was already reported previously [10], can be eliminated. The significant increase in shoot length was observed only in CNF-treated plants, whereas the root length in CO-treated plants was higher than in the other groups (Figure 1a). No biomass increase was observed both in the examined shoots and roots, because of the absence of exogenous nutrients (Figure 1b).

### 2.2. Comparison and Analysis of Differentially Expressed Genes (DEGs) in Chitin-treated Soybeans

**Figure 2:**
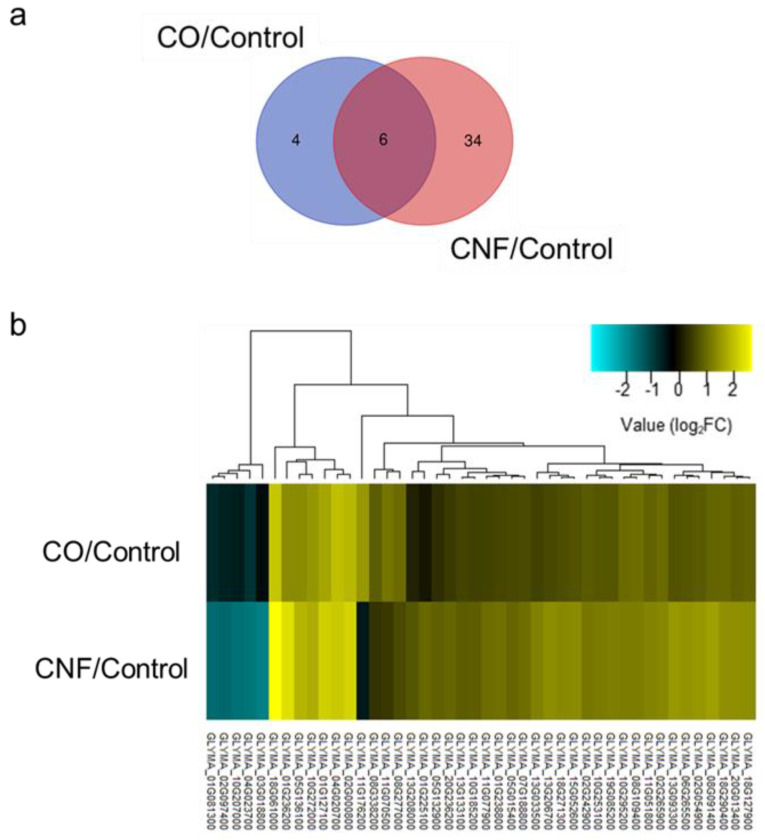
Differential expression analysis in chitin oligosaccharides (CO)- and chitin nanofiber (CNF)-treated soybean plants. Venn diagram (**a**) and clustered heatmap (**b**) of all differentially expressed genes.

Only CNF promoted the growth of aerial parts in soybeans under no exogenous nutrient supplement, which suggests that exclusively chitin-treated plants may be a better option to examine the differences in the effects of CO and CNF. Transcriptome analysis through RNA-seq of soybeans exclusively chitin-treated plants was conducted. The summary of sequencing, including the number (ca. 5.2–9.5M) and mapping efficiency (ca. 85–90%), to reference the genome sequence of raw reads for three biological replicates of each treatment is shown in Table S1. After the expression profile comparison between control and chitin-treated soybeans, 10 and 40 genes showed significantly different expression levels in CO- and CNF-treated plants, respectively (false discovery rate [FDR] cutoff < 0.05) (Table 1). Of these, four genes with annotations of syringolide-induced protein 14-1-1 (GLYMA_04G020700), glutamate decarboxylase (GLYMA_05G136100), naringenin-chalcone synthase 1 (GLYMA_08G109400), and C2H2-type domain-containing protein (GLYMA_10G295200) and two genes without annotations were determined as common DEGs in both chitin treatments (Figure 2a, Table 1). Next, 44 genes determined as DEGs in CO- and/or CNF-treated plants were clustered based on fold change values, as compared with control (Figure 2b). The clustered heatmap clearly showed that the expression patterns (upregulation or downregulation) of all DEGs were mostly similar in both chitin treatments, but DEGs expression observed in CNF-treated plants was strongly induced by CNF treatment, as compared with CO. Only DEGs in CNF-treated plants were further analyzed since the DEGs level in the CO-treated plants was not enough for functional analysis.

All DEGs in CNF-treated plants were subjected to gene ontology (GO) analysis to obtain deep functional characterization. The number of DEGs categorized by GO terms is shown in Table 2. The dominant number of GO terms were the following: “cell periphery” (GO:0071944) and “plasma membrane” (GO:0005886) in the cellular component category; “drug binding” (GO:0008144), “transporter activity” (GO:0005215), “localization” (GO:0051179), “establishment of localization” (GO:0051234), and “transmembrane transporter activity” (GO:0022857) in the molecular function category; and “response to stimulus” (GO:0050896) in the biological process category.

Next, GO enrichment analysis and Kyoto Encyclopedia of Genes and Genomes (KEGG) pathway analysis of these DEGs were performed to explore the potential biological processes and pathways that contribute to CNF function using ShinyGO with the FDR value cutoff of 0.05 [12]. There were 25 and 5 significantly overrepresented GO terms and KEGG pathways, respectively (Table 3). The genes involved in “carbohydrate transport” (GO:0008643), “glutamate catabolic process” (GO:0006538), “drug binding” (GO:0008144), “calcium ion binding” (GO:0005509), “peroxidase activity” (GO:0004601), “butanoate metabolism” (KEGG:map00650), and “MAPK (mitogen-activated protein kinase) signaling pathway” (KEGG:map04010) were significantly induced by CNF supplementation, considering the results of hierarchical trees summarizing significant GO terms and KEGG pathways (Figures S1 and S2).

## 3. Discussion

In this study, we showed that CNF supplementation to soils, rather than CO, promoted the growth of aerial parts and induced a stronger plant response to chitin. This is the first report that directly and comprehensively compared the effects of high- and low-molecular-weight chitins on plants by transcriptome analysis using RNA-seq. In a previous study [10], both CO and CNF showed growth-promoting action on the aerial parts of tomato by improving nitrogen uptake efficiency, which is inconsistent with the results of promoting effects on soybean growth found in this study. The transcriptome analysis of CNF-treated tomato indicated that the expression levels of genes related to nitrogen acquisition and assimilation and nutrient allocation were changed [10]. As previously described in this study, the effects of chitin treatments on soybean growth without exogenous nutrient application were examined, which eliminates the influence of nitrogen acquisition. Therefore, the inconsistency on the growth-promoting action by both chitins may be caused by the CNF-specific function involved in the regulation of nitrogen assimilation and nutrient allocation. However, the transcriptome analysis in this study demonstrated that all the DEGs’ expression patterns were mostly similar in both chitin treatments, but DEGs expression was strongly induced by CNF treatment, as compared with CO treatment. This indicates that both CO and CNF show a mode of action that is common in plants, but the degree of plant response to chitin by CNF is stronger than by CO. This is also supported by the evidence that CNF induced more ROS production than CO in *Arabidopsis*, but ROS production was completely impaired in chitin receptor CERK1 mutant *cerk1-2* [5,13]. We, therefore, concluded that the growth-promoting effects on the aerial parts of soybeans by CNF would not be caused by CNF-specific function and may be attributed to the different levels of plant response to chitin caused by CO and CNF.

In plants, chitin is recognized by the ectodomains of LysM (lysin motif) receptors, such as CEBiP and CERK1, localized on plasma membranes, and then chitin recognition information is transduced by LysM receptor phosphorylation to activate downstream signaling [4,13,14]. *Arabidopsis* CERK1 binds to polymeric chitin and plays an essential role in chitin signaling [15,16], and CNF is also recognized by CERK1 in *Arabidopsis* similar to CO, because of the lack of ROS production by CNF in *cerk1-2* [5], suggesting that the primary site of action by CNF is in the apoplast, similar to CO. The molecular basis that explains the reason why CNF can induce stronger plant response to chitin than CO is still unclear because it has not been clarified whether CNF directly binds to CERK1. The rapid and massive CO production by chitinase presented or secreted from plant cells in the apoplast may cause CNF’s stronger induction since CNF can be immediately degraded to CO by chitinase [5].

The GO analysis of DEGs in CNF-treated plants showed that most of the genes differentially expressed by CNF treatment in soybeans may be involved in the function in the apoplast and the communication between inner and outer plasma membranes according to the categorized GO terms like “cell periphery,” “plasma membrane,” “transporter activity,” and “response to stimulus,” which is consistent with the aforementioned possible primary site of CNF action. The GO enrichment analysis and KEGG pathway analysis of DEGs in CNF-treated plants demonstrated the potential biological functions of chitins because the mode of action by CO and CNF in plants is common. The overrepresented GO terms “carbohydrate transport” and “glutamate catabolic process” may be associated with the growth-promoting effects of chitins. This is consistent with previous findings of increased carbon content by both CO and CNF treatments associated with its growth-promoting action, and the genes involved in glutamate catabolic process, such as glutamate synthase and glutamate dehydrogenase, were upregulated in CNF-treated tomato [10]. The genes involved in the “butanoate metabolism” pathway are also overrepresented. In this metabolic pathway, a non-protein amino acid, gamma-aminobutyric acid (GABA), is primarily biosynthesized from glutamate by glutamate decarboxylase in plants, which modulates plant growth [17,18]. The gene encoding glutamate decarboxylase (GLYMA_05G136100) was upregulated by both CO and CNF treatments, which suggests that the function of GABA is required for growth promotion in chitin-treated plants. Moreover, the overrepresented GO terms “calcium ion binding,” “peroxidase activity,” and KEGG-related “MAPK signaling pathway” seem to be associated with chitin-induced defense response, because MAMPs, including chitin, quickly induce Ca^2+^ influx, expression of peroxidase, ROS production, and MAPK signaling activation in plants [19,20]. Chitin rapidly upregulated genes encoding NADPH oxidases, which are involved in ROS production on plasma membrane, *rbohD* and *RbohF* in *Arabidopsis* [21]. Likewise, the expression level of gene (GLYMA_20G236200) encoding unknown protein homologous to NADPH oxidase was increased by both CO and CNF treatments in soybeans.

In summary, we demonstrated that nanofibrillation, rather than generally oligomerization, is an effective method to produce chitin derivatives to induce the plant response in soybeans. To develop ecofriendly chitin derivatives that are agriculturally usable, several studies on applying chitin and chitosan nanoparticles have recently been reported [22,23]. However, the production cost of these nanoparticles may be more than CNF, which only demands machines, such as a grinder, for physical treatment because these nanoparticles still demand the usage of chemicals [2,24]. Considering not only the advantage of biological function but also its unique physical features and chitin production without hazardous chemicals, nanofibrillation is the best choice to develop practical and promising ecofriendly chitin derivatives, which can be agriculturally applied to improve crop yield. Chemicals are not required for CNF production from original chitin, but hazardous chemicals are necessary to produce original chitins from raw materials, such as crab and shrimp shells. The nanofibrillation technique of raw materials can be used, so that using chemicals during the whole process can be avoided, since CNF composites prepared directly from crab shells showed similar effects on disease resistance in tomato, as compared with pure CNF [7]. The knowledge obtained in this study is not restricted to soybeans but is also expected to be applicable to other plant species, because the functions of CNF that contributed to crop yield improvement, such as growth promotion and induction of disease resistance, have already been confirmed in various plant species [5,6,7,10].

## 4. Materials and Methods

### 4.1. Preparation of Chitin Nanofiber and Chitin Oligosaccharide Water Dispersions

CNF homogeneous water dispersion was prepared from chitin powder derived from crab shells (chitin TC-L; procured from Koyo Chemical, Japan) without acetic acid, as previously described [25]. Furthermore, dry chitin powder was mixed in distilled water at 1 wt.% and passed through a grinder (MKZA12-20J; Masuko Sangyo, Japan.) for two cycles to obtain CNF water dispersion. CO solution at 1 wt.% was prepared by dissolving CO powder mixture (NA-COS-Y; purchased from Yaizu Suisankagaku Industry, Japan), which consists of Cos with DP2–6 [5], in distilled water.

### 4.2. Plant Materials, Growth Conditions, and Measurement of Plant Growth

The surface-sterilized soybean (*Glycine max* cv. Enrei) seeds were grown on sterilized soil (1:2 mixture of vermiculite and river sand) mixed with an equal volume of 0.01% (*w/v*) CO solution or CNF dispersion, or water as a control, under a cool white fluorescent lamp (50 μmol m^−2^ s^−1^, 14 h light/10 h dark cycle) at 24 °C for 4 weeks. For growth measurement, the length of harvested shoots and roots was measured and subjected to oven-drying at 60 °C until completely dried, and then dry weight was measured. The significant difference in each measurement was determined using Tukey’s HSD test.

### 4.3. RNA Sequencing and Data Analysis

Soybean plants were grown on the moist vermiculite for 1 week after germination. Then, 1-week-old seedlings were washed with water and then immersed in 0.01% (*w/v*) CO solution or CNF dispersion, or water as a control. After shaking gently for 1 h, three plants were collected for each repetition per treatment. Total RNA was extracted from three different pools using an RNeasy Plant Kit (Qiagen, Netherlands). RNA-seq libraries were prepared from 0.5 µg of the total RNA using TruSeq RNA sample preparation kit (Illumina, San Diego, CA, USA) according to the manufacturer’s instructions. The library quality was assessed using the Agilent DNA 1000 kit and the 2100 Bioanalyzer system. The libraries were sequenced with single-end 100 bp reads on the Hiseq 1500 sequencing system (Illumina). The acquired sequences were mapped to the reference genome of *G. max* v2.1 obtained from Ensembl Plants (http://plants.ensembl.org/Glycine_max/Info/Index) using STAR v2.6.1 [26] with default parameters. Read counts per gene were quantified using featureCounts v1.6.5 [27]. The expression profiles were compared between control and chitin-treated seedlings using edgeR with trimmed mean of M values for normalization [28], and then the lists of DEGs were obtained (FDR cutoff < 0.05). GO enrichment and KEGG pathway analyses (FDR cutoff < 0.05) of DEGs were conducted using ShinyGO v0.61 (http://bioinformatics.sdstate.edu/go/) [12]. Raw nucleotide sequence data are available from the DDBJ Sequence Read Archive under accession number DRA008927.

## Figures and Tables

**Table 1:** Significantly differentially expressed genes (DEGs) in chitin oligosaccharides (CO) and/or chitin nanofiber (CNF)-treated soybean plants.

Gene ID	Description	CO/Control		CNF/Control	
		log2FC	FDR ^a^	log2FC	FDR ^a^
CO only					
GLYMA_11G176200	Uncharacterized protein	1.76	2.74 $\times \;10^{-4}$	−0.31	1.00 $\times \;10^{0}$
GLYMA_11G070500	Isoflavone reductase, NmrA domain-containing protein	1.36	1.98 $\times \;10^{-4}$	0.67	9.43 $\times \;10^{-1}$
GLYMA_08G277000	TRANSKETOLASE_1 domain-containing protein	1.26	1.17 $\times \;10^{-2}$	0.88	3.36 $\times \;10^{-1}$
GLYMA_08G338200	Glycosyltransferase (EC 2.4.1.-)	1.09	3.04 $\times \;10^{-2}$	0.62	1.00 $\times \;10^{0}$
CO and CNF					
GLYMA_04G020700	Syringolide-induced protein 14-1-1	2.22	1.47 $\times \;10^{-4}$	2.34	7.60 $\times \;10^{-4}$
GLYMA_10G272000	Uncharacterized protein	1.74	1.99 $\times \;10^{-2}$	2.00	4.01 $\times \;10^{-2}$
GLYMA_05G136100	Glutamate decarboxylase (EC 4.1.1.15)	1.63	2.30 $\times \;10^{-3}$	2.11	6.65 $\times \;10^{-4}$
GLYMA_08G109400	Chalcone synthase 1 (EC 2.3.1.74)	1.30	7.11 $\times \;10^{-3}$	1.45	1.79 $\times \;10^{-2}$
GLYMA_10G295200	C2H2-type domain-containing protein	1.25	1.50 $\times \;10^{-2}$	1.47	3.87 $\times \;10^{-2}$
GLYMA_02G265900	Uncharacterized protein	1.24	5.89 $\times \;10^{-4}$	1.57	2.97 $\times \;10^{-4}$
CNF only					
GLYMA_18G061000	PPM-type phosphatase domain-containing protein	2.28	2.42 $\times \;10^{-1}$	2.98	3.85 $\times 10^{-2}$
GLYMA_01G236200	Uncharacterized protein	1.61	1.00 $\times \;10^{0}$	2.59	3.20 $\times \;10^{-3}$
GLYMA_02G000800	BHLH domain-containing protein	2.13	5.36 $\times \;10^{-2}$	2.46	1.51 $\times \;10^{-2}$
GLYMA_01G127100	Dirigent protein	1.91	6.91 $\times \;10^{-2}$	2.42	5.28 $\times \;10^{-3}$
GLYMA_08G091400	Glutamate decarboxylase (EC 4.1.1.15)	1.18	2.42 $\times \;10^{-1}$	1.86	7.46 $\times \;10^{-3}$
GLYMA_13G093300	PBPe domain-containing protein	0.96	5.93 $\times \;10^{-1}$	1.76	1.79 $\times \;10^{-3}$
GLYMA_02G054900	Uncharacterized protein	1.06	1.00 $\times \;10^{0}$	1.76	1.42 $\times \;10^{-2}$
GLYMA_06G265500	GRAS domain-containing protein	1.01	1.47 $\times \;10^{-1}$	1.71	1.37 $\times \;10^{-4}$
GLYMA_20G013400	Uncharacterized protein	1.11	9.36 $\times \;10^{-1}$	1.67	1.82 $\times 10^{-3}$
GLYMA_18G290400	Uncharacterized protein	1.19	1.00 $\times \;10^{0}$	1.66	2.68 $\times \;10^{-2}$
GLYMA_18G127900	TIR-NBS-LRR type disease resistance protein	1.14	5.75 $\times \;10^{-1}$	1.65	1.79 $\times \;10^{-2}$
GLYMA_13G206700	TPT domain-containing protein	0.85	8.68 $\times \;10^{-1}$	1.65	5.62 $\times \;10^{-6}$
GLYMA_15G052600	Peroxidase (EC 1.11.1.7)	0.95	1.00 $\times \;10^{0}$	1.63	8.57 $\times \;10^{-3}$
GLYMA_18G271300	Protein kinase domain-containing protein	0.93	2.41 $\times \;10^{-1}$	1.59	2.18 $\times \;10^{-4}$
GLYMA_11G051800	Uncharacterized protein	1.16	5.82 $\times \;10^{-1}$	1.53	1.49 $\times \;10^{-3}$
GLYMA_13G033500	Protein kinase domain-containing protein	0.78	1.00 $\times \;10^{0}$	1.50	2.55 $\times \;10^{-2}$
GLYMA_19G085200	Uncharacterized protein	1.01	9.23 $\times \;10^{-1}$	1.47	8.31 $\times \;10^{-4}$
GLYMA_10G253100	Uncharacterized protein	1.01	3.80 $\times \;10^{-1}$	1.43	3.85 $\times \;10^{-2}$
GLYMA_02G242900	RING-type E3 ubiquitin transferase (EC 2.3.2.27)	1.06	5.80 $\times \;10^{-1}$	1.39	3.87 $\times \;10^{-2}$
GLYMA_01G238800	MFS domain-containing protein	0.81	7.33 $\times \;10^{-1}$	1.30	7.50 $\times \;10^{-3}$
GLYMA_07G188800	Uncharacterized protein	0.88	2.42 $\times \;10^{-1}$	1.28	5.87 $\times \;10^{-3}$
GLYMA_11G077900	Rhodanese domain-containing protein	0.77	7.06 $\times \;10^{-1}$	1.27	1.42 $\times 10^{-2}$
GLYMA_01G225100	PPM-type phosphatase domain-containing protein	0.28	1.00 $\times \;10^{0}$	1.23	3.46 $\times 10^{-2}$
GLYMA_05G015400	Phospholipid-transporting ATPase (EC 7.6.2.1)	0.85	2.42 $\times \;10^{-1}$	1.20	3.20 $\times 10^{-3}$
GLYMA_20G236200	Uncharacterized protein	0.68	1.00 $\times \;10^{0}$	1.19	4.31 $\times 10^{-3}$
GLYMA_05G132900	Uncharacterized protein	0.55	1.00 $\times \;10^{0}$	1.12	3.87 $\times 10^{-2}$
GLYMA_10G185200	Zeta_toxin domain-containing protein	0.77	5.92 $\times \;10^{-1}$	1.12	3.87 $\times 10^{-2}$
GLYMA_13G133100	Uncharacterized protein	0.79	5.47 $\times \;10^{-1}$	1.08	4.80 $\times 10^{-2}$
GLYMA_13G208000	Peptidase A1 domain-containing protein	0.41	1.00 $\times \;10^{0}$	1.00	1.42 $\times 10^{-2}$
GLYMA_02G097400	Protein kinase domain-containing protein	−0.42	1.00 $\times \;10^{0}$	−1.28	2.68 $\times 10^{-2}$
GLYMA_01G081300	Uncharacterized protein	−0.50	1.00 $\times \;10^{0}$	−1.28	1.88 $\times 10^{-2}$
GLYMA_10G207000	Uncharacterized protein	−0.35	1.00 $\times \;10^{0}$	−1.38	1.79 $\times 10^{-2}$
GLYMA_04G023700	Uncharacterized protein	−0.61	1.00 $\times \;10^{0}$	−1.43	1.49 $\times 10^{-2}$
GLYMA_03G018800	TCP domain-containing protein	−0.15	1.00 $\times \;10^{0}$	−1.56	3.85 $\times 10^{-2}$

^a^ False discovery rate: DEGs were determined using FDR (< 0.05).

**Table 2:** The number of differentially expressed genes categorized by gene ontology (GO) terms in chitin nanofiber-treated soybean plants.

GO Term	Category ^a^	N	Genes
Drug binding	MF	10	GLYMA_02G097400 GLYMA_05G015400 GLYMA_05G132900 GLYMA_05G136100 GLYMA_07G188800 GLYMA_08G091400 GLYMA_10G185200 GLYMA_10G253100 GLYMA_13G033500 GLYMA_18G271300
Cell periphery	CC	9	GLYMA_01G238800 GLYMA_02G097400 GLYMA_05G015400 GLYMA_07G188800 GLYMA_10G253100 GLYMA_13G093300 GLYMA_15G052600 GLYMA_20G013400 GLYMA_20G236200
Plasma membrane	CC	8	GLYMA_01G238800 GLYMA_02G097400 GLYMA_05G015400 GLYMA_07G188800 GLYMA_10G253100 GLYMA_13G093300 GLYMA_20G013400 GLYMA_20G236200
Transporter activity	MF	6	GLYMA_01G238800 GLYMA_05G015400 GLYMA_05G132900 GLYMA_13G093300 GLYMA_13G206700 GLYMA_19G085200
Response to stimulus	BP	5	GLYMA_13G093300 GLYMA_13G133100 GLYMA_15G052600 GLYMA_18G127900 GLYMA_20G236200
Localization	BP	5	GLYMA_01G238800 GLYMA_05G015400 GLYMA_05G132900 GLYMA_13G206700 GLYMA_19G085200
Establishment of localization	BP	5	GLYMA_01G238800 GLYMA_05G015400 GLYMA_05G132900 GLYMA_13G206700 GLYMA_19G085200
Transmembrane transporter activity	MF	5	GLYMA_01G238800 GLYMA_05G132900 GLYMA_13G093300 GLYMA_13G206700 GLYMA_19G085200
Catabolic process	BP	4	GLYMA_05G136100 GLYMA_08G091400 GLYMA_13G208000 GLYMA_15G052600
Cellular response to stimulus	BP	4	GLYMA_13G093300 GLYMA_15G052600 GLYMA_18G127900 GLYMA_20G236200
Cofactor binding	MF	4	GLYMA_05G136100 GLYMA_08G091400 GLYMA_11G051800 GLYMA_15G052600
Regulation of metabolic process	BP	3	GLYMA_03G018800 GLYMA_06G265500 GLYMA_10G295200
Response to chemical	BP	3	GLYMA_13G133100 GLYMA_15G052600 GLYMA_20G236200
DNA-binding transcription factor activity	MF	3	GLYMA_03G018800 GLYMA_06G265500 GLYMA_10G295200
Oxidoreductase activity	MF	3	GLYMA_11G051800 GLYMA_15G052600 GLYMA_20G236200
Signaling	BP	2	GLYMA_13G093300 GLYMA_18G127900
Detoxification	BP	2	GLYMA_15G052600 GLYMA_20G236200
Cellular detoxification	BP	2	GLYMA_15G052600 GLYMA_20G236200
Extracellular region	CC	2	GLYMA_01G127100 GLYMA_15G052600
Cell-cell junction	CC	2	GLYMA_02G097400 GLYMA_15G052600
Endomembrane system	CC	2	GLYMA_05G015400 GLYMA_13G206700
Cell junction	CC	2	GLYMA_02G097400 GLYMA_15G052600
Symplast	CC	2	GLYMA_02G097400 GLYMA_15G052600
Peroxidase activity	MF	2	GLYMA_15G052600 GLYMA_20G236200
Antioxidant activity	MF	2	GLYMA_15G052600 GLYMA_20G236200
Lyase activity	MF	2	GLYMA_05G136100 GLYMA_08G091400
Carbohydrate binding	MF	2	GLYMA_01G081300 GLYMA_13G033500

^a^ BP: Biological Process, CC: Cellular Component, MF: Molecular Function.

**Table 3:** Gene ontology (GO) enrichment analysis and Kyoto Encyclopedia of Genes and Genomes (KEGG) pathway analysis of differentially expressed genes in chitin nanofiber-treated soybean plants.

Functional Category (GO Category ^a^ or KEGG Pathway)	Genes in List	Total Genes	Enrichment FDR ^b^
Glutamate catabolic process (BP)	2	10	1.68 $\times \;10^{-3}$
Glutamate decarboxylase activity (MF)	2	10	1.68 $\times \;10^{-3}$
Dicarboxylic acid catabolic process (BP)	2	13	1.94 $\times \;10^{-3}$
Taurine and hypotaurine metabolism (KEGG)	2	29	6.43 $\times \;10^{-3}$
Glutamine family amino acid catabolic process (BP)	2	30	6.43 $\times \;10^{-3}$
Butanoate metabolism (KEGG)	2	33	6.50 $\times \;10^{-3}$
Glutamate metabolic process (BP)	2	37	7.01 $\times \;10^{-3}$
Plasma membrane (CC)	8	2728	9.11 $\times \;10^{-3}$
Cell periphery (CC)	9	3371	9.11 $\times \;10^{-3}$
MAPK signaling pathway (KEGG)	3	230	9.11 $\times \;10^{-3}$
Alanine, aspartate and glutamate metabolism (KEGG)	2	73	1.73 $\times \;10^{-2}$
Beta-Alanine metabolism (KEGG)	2	81	1.95 $\times \;10^{-2}$
Drug binding (MF)	10	4868	2.12 $\times \;10^{-2}$
Alpha-amino acid catabolic process (BP)	2	110	3.05 $\times \;10^{-2}$
Carboxy-lyase activity (MF)	2	119	3.17 $\times \;10^{-2}$
Cellular amino acid catabolic process (BP)	2	120	3.17 $\times \;10^{-2}$
Organophosphate ester transport (BP)	2	130	3.23 $\times \;10^{-2}$
Carbohydrate transmembrane transport (BP)	2	139	3.23 $\times \;10^{-2}$
Dicarboxylic acid metabolic process (BP)	2	142	3.23 $\times \;10^{-2}$
Magnesium-dependent protein serine/threonine phosphatase activity (MF)	2	134	3.23 $\times \;10^{-2}$
Calcium ion binding (MF)	3	474	3.23 $\times \;10^{-2}$
Glutamine family amino acid metabolic process (BP)	2	137	3.23 $\times \;10^{-2}$
Metal ion binding (MF)	9	4694	3.35 $\times \;10^{-2}$
Carbohydrate transmembrane transporter activity (MF)	2	156	3.38 $\times \;10^{-2}$
Cation binding (MF)	9	4733	3.38 $\times \;10^{-2}$
Carbon-carbon lyase activity (MF)	2	173	3.82 $\times \;10^{-2}$
Carbohydrate transport (BP)	2	170	3.82 $\times \;10^{-2}$
Organic acid catabolic process (MF)	2	191	4.18 $\times \;10^{-2}$
Carboxylic acid catabolic process (MF)	2	191	4.18 $\times \;10^{-2}$
Transporter activity (MF)	6	2526	4.18 $\times \;10^{-2}$

^a^ BP: Biological Process, CC: Cellular Component, MF: Molecular Function; ^b^ False discovery rate (cutoff <0.05).

## Supplementary Materials

The following are available online at https://www.mdpi.com/2223-7747/9/7/810/s1, Figure S1: Hierarchical trees summarizing significant gene ontology (GO) terms, Figure S2: Hierarchical tree summarizing significant Kyoto Encyclopedia of Genes and Genomes (KEGG) pathways, Table S1: Summary of transcriptome analysis.
