# Supplementary material for: Nanofibrillation is an Effective Method to Produce Chitin Derivatives for Induction of Plant Responses in Soybean
Source: Plants (Basel). 2020 Jun 28;9(7):810. doi: 10.3390/plants9070810 (PMC7411678; doi:10.3390/plants9070810)
Supplement: Supplementary file 1 [file plants-09-00810-s001.pdf]

a

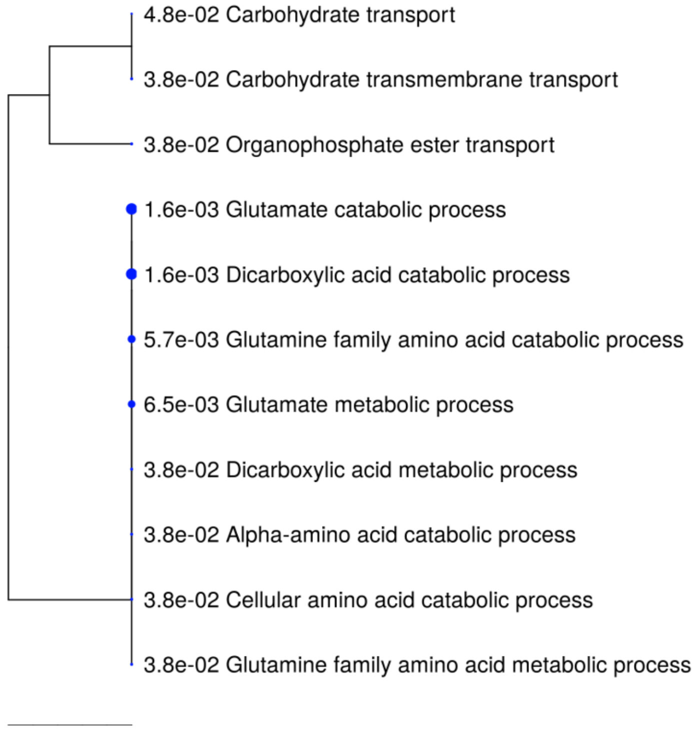

b

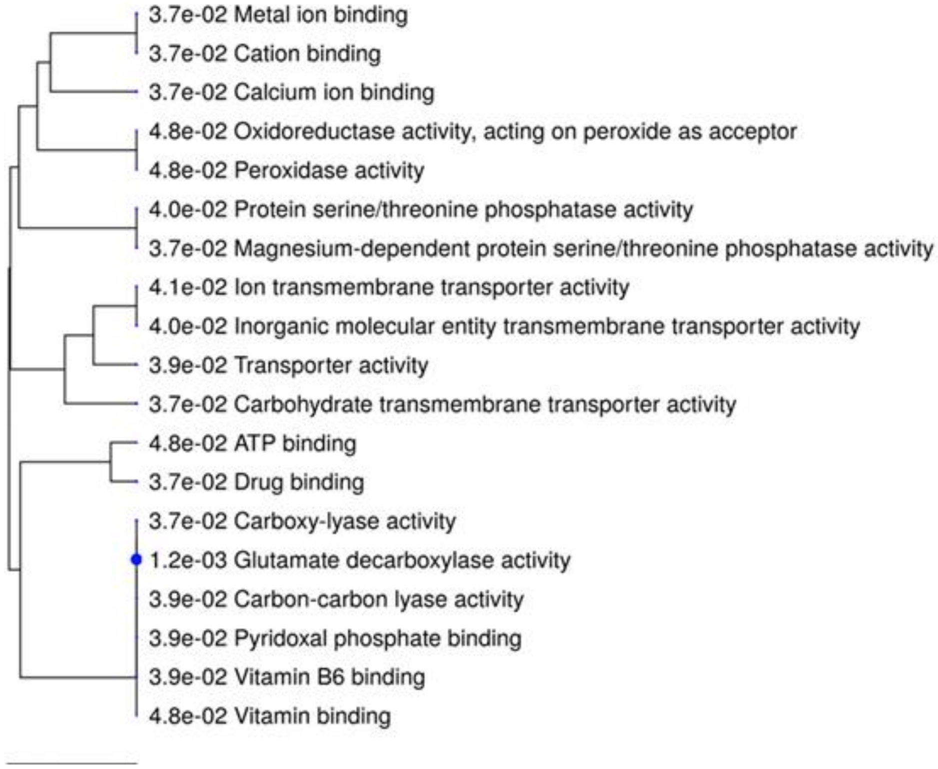

Supplemental Figure S1. Hierarchical trees summarizing significant gene ontology (GO) terms. The trees of GO categories, Biological Process (a) and Molecular Function (b), were generated by ShinyGO web site. GO Terms sharing more genes are grouped, and bigger dots at the end of branches correspond to more significant FDR values.

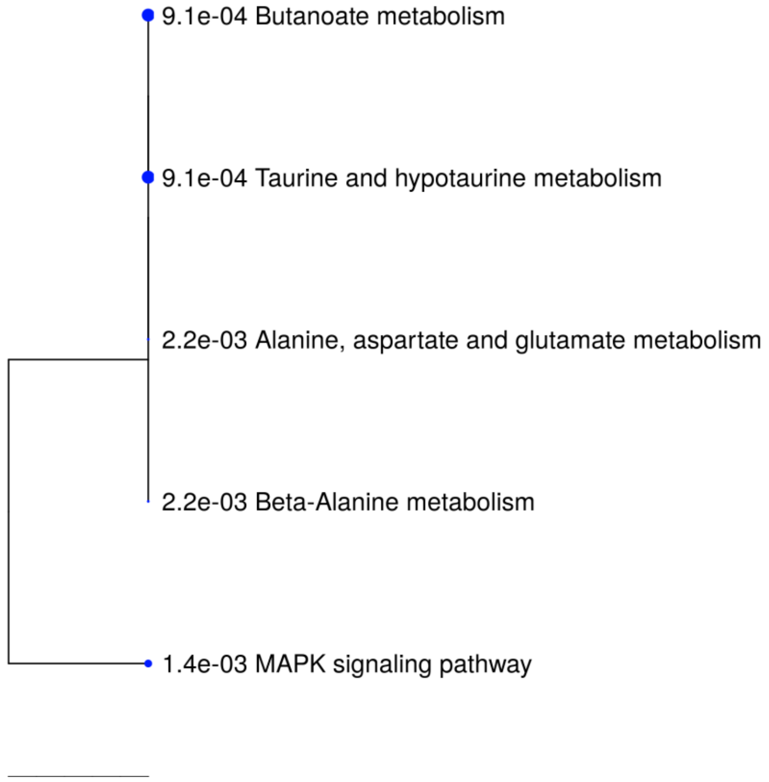

Supplemental Figure S2 Hierarchical tree summarizing significant Kyoto Encyclopedia of Genes and Genomes (KEGG) pathways. The details of tree are found in Fig. S1.

Table S1 Summary of transcriptome analysis

Sample name	Control-1	Control-2	Control-3	CO-1	CO-2	CO-3	CNF-1	CNF-2	CNF-3
Number of raw reads	5197979	6750046	9538029	8516607	7100973	5829062	7427079	7399271	6922205
Average read length (bp)	126	126	126	126	126	126	126	126	126
Uniquely mapped reads	92.54%	76.17%	93.00%	93.02%	92.77%	92.28%	92.34%	91.96%	91.14%
Average mapped length (bp)	125.04	123.45	124.89	125.07	124.95	125.01	124.9	124.99	125.04
Successfully assigned alignments	89.10%	85.10%	89.60%	89.20%	89.20%	89.60%	89.50%	89.70%	89.40%
